# Vitamin C sensitizes triple negative breast cancer to PI3K inhibition therapy

**DOI:** 10.7150/thno.53225

**Published:** 2021-01-20

**Authors:** Sushmita Mustafi, Vladimir Camarena, Rehana Qureshi, David W. Sant, Zachary Wilkes, Daniel Bilbao, Joyce Slingerland, Susan B. Kesmodel, Gaofeng Wang

**Affiliations:** 1 John P. Hussman Institute for Human Genomics, Dr. John T. Macdonald Foundation Department of Human Genetics, University of Miami Miller School of Medicine, Miami, Florida.; 2 Sylvester Comprehensive Cancer Center, University of Miami Miller School of Medicine, Miami, Florida.; 3 Braman Family Breast Cancer Institute at Sylvester, University of Miami Miller School of Medicine, Miami, Florida.

## Abstract

**Rationale:** The clinical use of PI3K inhibitors, such as buparlisib, has been plagued with toxicity at effective doses. The aim of this study is to determine if vitamin C, a potent epigenetic regulator, can improve the therapeutic outcome and reduce the dose of buparlisib in treating *PIK3CA*-mutated triple negative breast cancer (TNBC).

**Methods:** The response of TNBC cells to buparlisib was assessed by EC_50_ measurements, apoptosis assay, clonogenic assay, and xenograft assay in mice. Molecular approaches including Western blot, immunofluorescence, RNA sequencing, and gene silencing were utilized as experimental tools.

**Results:** Treatment with buparlisib at lower doses, along with vitamin C, induced apoptosis and inhibited the growth of TNBC cells *in vitro*. Vitamin C via oral delivery rendered a sub-therapeutic dose of buparlisib able to inhibit TNBC xenograft growth and to markedly block metastasis in mice. We discovered that buparlisib and vitamin C coordinately reduced histone H3K4 methylation by enhancing the nuclear translocation of demethylase, KDM5, and by serving as a cofactor to promote KDM5-mediated H3K4 demethylation. The expression of genes in the PI3K pathway, such as AKT2 and mTOR, was suppressed by vitamin C in a KDM5-dependent manner. Vitamin C and buparlisib cooperatively blocked AKT phosphorylation. Inhibition of KDM5 largely abolished the effect of vitamin C on the response of TNBC cells to buparlisib. Additionally, vitamin C and buparlisib co-treatment changed the expression of genes, including PCNA and FILIP1L, which are critical to cancer growth and metastasis.

**Conclusion:** Vitamin C can be used to reduce the dosage of buparlisib needed to produce a therapeutic effect, which could potentially ease the dose-dependent side effects in patients.

## Introduction

In human breast cancer, the most frequently mutated gene is *PIK3CA*, which is seen in ~30% of cases 1, 2. *PIK3CA* mutations have been identified across the spectrum of subtypes, including hormone receptor positive (HR+), human epidermal growth factor receptor 2 positive (HER2+), and triple negative 3-5. *PIK3CA* encodes the PI3Kα protein, a key upstream component of the PI3K pathway which transduces the signaling of receptors, including HER2, and controls a variety of essential cellular processes. Clustered at the helical and kinase domains, *PIK3CA* mutations increase the catalytic activity of PI3K resulting in enhanced downstream signaling, which further promotes cell proliferation, survival and migration, contributing to oncogenic transformation 6, 7. Other genes in the PI3K pathway, such as *PTEN*, *AKT1* and *PIK3R1*, are also mutated in breast cancer but with much lower frequency. Overall, the discovery of highly frequent activating mutations in *PIK3CA,* and the appreciation of its role as a key driving factor of breast oncogenesis, has greatly improved our understanding of the disease.

Targeting the dysregulated PI3K pathway, especially the mutant PI3K, has long been recognized as a potential treatment for breast cancer 8. Various PI3K inhibitors, including pan-PI3K inhibitors and PI3Kα specific inhibitors, have been developed and tested. Numerous preclinical studies have shown that PI3K inhibition can effectively treat breast cancer in model systems 9. However, the clinical results of PI3K inhibitors, in combination with endocrine therapy or anti-HER2 therapy, have unfortunately been modest. It appears that PI3K inhibitors are plagued with a relatively narrow therapeutic window as manifested by low responses, a mild decrease in disease progression, and by dose-dependent toxicities, such as hyperglycemia and diarrhea 10-13. The obvious obstacle preventing these promising cancer drugs from being utilized for clinical patient care is the narrow therapeutic window. If an agent that is able to sensitize breast cancer to PI3K inhibitors is identified and applied, lower doses of these inhibitors could then be used to achieve an increased therapeutic index in patients, which will also translate to a reduced side effect profile in patients.

Genetic and epigenetic variations contribute to drug responses 14. Epigenetic changes are reversible and therefore provide an opportunity to improve drug response by targeting relevant epigenetic regulators. We and others have shown that vitamin C promotes demethylation of DNA 15-18 and histones 19, 20 by its capacity to convert catalytically inactive Fe(III) to active Fe(II), which is an essential cofactor for ten-eleven translocation (TET) methylcytosine dioxygenases and JmjC domain-containing histone demethylases. Given the proven role of vitamin C in epigenetic regulation, we reasoned that bioavailability of vitamin C may influence drug responses. Vitamin C enters and accumulates in breast epithelial cells mainly through sodium-dependent vitamin C transporter 2 (SVCT2) 21. Our earlier work discovered that SVCT2 expression was decreased in breast cancers compared to normal breast tissues from the same patients 22. Higher doses of vitamin C may be required to compensate for the downregulated SVCT2 in breast cancer.

Unlike HR+ or HER2+ subtypes, triple negative breast cancer (TNBC), which is often associated with a more aggressive clinical course and poor survival, still lacks targeted therapy. About 20% of TNBC cases carry *PIK3CA* mutations 23. Without HER2 overexpression, the mutated *PIK3CA* is the major driver for the overactivated PI3K signaling in TNBC, suggesting that PI3K inhibition could be a potential targeted therapy for TNBC. This study investigated if the response of *PIK3CA*-mutated TNBC to PI3K inhibitor could be improved by vitamin C. Here, we show that vitamin C at 100 μM, which is achievable in the plasma *in vivo* by oral delivery, markedly increases the efficacy of PI3K inhibitor buparlisib in treating TNBC cells. Oral vitamin C supplementation improves the response of TNBC xenografts to buparlisib in mice. An epigenetic mechanism, centered on histone demethylase KDM5, predominantly underpins the improved response of TNBC to buparlisib by vitamin C. Buparlisib and vitamin C coordinately promote KDM5-mediated histone H3K4 demethylation by increasing the nuclear translocation of KDM5 and by serving as a cofactor to promote KDM5-mediated demethylation. Subsequently, the expression of genes, especially the ones in the PI3K pathway and the ones critical to cancer growth and metastasis, are altered by buparlisib and vitamin C cooperatively. Overall, the sensitization of TNBC to buparlisib by vitamin C is largely mediated by KDM5. Since vitamin C is a safe and well-tolerated micronutrient, this work supports a practical translation to the clinical setting to test if vitamin C can improve the benefit/toxicity ratio of PI3K inhibitors in treating patients with *PIK3CA*-mutated TNBC.

## Methods

### Cell culture and treatment

TNBC cell lines including BT20 and MDA-MB-453, which both carry activating *PIK3CA* mutations, were purchased from ATCC (Manassas, VA) in the year of 2018 without further authentication. Frozen cells were newly thawed from low (2-5) passages. Testing for mycoplasma was performed using PlasmoTest Mycoplasma detection kits (Thermo Fisher, Waltham, MA) with only mycoplasma negative cells included in all experiments. BT20 cells were maintained under a 5% CO_2_ atmosphere in Eagle's Minimum Essential Medium (EMEM), while MDA-MB-453 cells were cultured in Leibovitz's L-15 Medium (ATCC), supplemented with 10% heat-inactivated fetal bovine serum, 100 Units/ ml penicillin, and 100 μg/ml of streptomycin. TNBC cells were seeded for 24 hours and subsequently treated with vitamin C (sodium ascorbate, Sigma-Aldrich, St. Louis, MO) and different PI3K inhibitors at various concentrations. Media was changed daily to ensure the presence of fresh vitamin C.

### EC_50_ measurement

BT20 and MDA-MB-453 cells were pretreated with or without vitamin C at various concentrations. On 384-well plates, a 3-fold serial dilution of buparlisib (also known as BKM120; Selleckchem, Houston, TX) was prepared with a starting concentration of 10 μM. On the day of treatment, buparlisib was simultaneously transferred to the cell plates with a 384-pipettor head using a FLIPR tetra instrument (Molecular Devices, Sunnyvale, CA). Cell viability was measured after 72 hours by CellTiter-GLO (Promega) assay. Each concentration of buparlisib was plotted against percent cell survival. The half maximal effective concentration (EC_50_) values were calculated from 4-parameter fitted curves by solving for the X-intercept value at the 50% inhibition level of the Y-intercept value.

### Apoptosis and clonogenic assays

For the apoptosis assay, TNBC cells were seeded in 24-well plates and pretreated with or without vitamin C (50, 100 μM) for 72 hours. Cells were then further treated with or without 0.5 μM buparlisib for an additional 48 hours. All treatments were conducted in triplicate. Apoptotic cells were determined by fluorescein-based TUNEL assay (Sigma-Aldrich) following the manufacturer's protocol. Clonogenic survival was defined by the ability of the cells to form colonies. Each colony contained at least 50 cells. Briefly, 500 cells were seeded into 6-well dishes in 2 mL medium. Two days later, different wells were treated with 0, 50 or 100 μM vitamin C alone or in conjunction with 0.5 μM of buparlisib. Plates were maintained for an additional 12 days with a daily treatment regimen of vitamin C and buparlisib treatment every 3 days. Cells were fixed with methanol-acetic acid (3:1) solution followed by crystal violet staining. The area of colonies per well was captured on a dissection microscope and analyzed. Each experiment was repeated in triplicate.

### Animal studies

All procedures were performed in accordance with guidelines approved by the Institutional Animal Care and Use Committee at the University of Miami. Female NOD scid gamma (NSG) mice were purchased from The Jackson Laboratory. BT20 (10^6^ cells) were engrafted on the 4^th^ mammary fat pad of each animal. Three weeks post implantation all animals obtained tumors around 100-150 mm^3^ volume measured by calipers. Mice were then randomly distributed into four groups of ~10 animals each. One group received vitamin C (3.3 g/L) in the water supply, one group received buparlisib (25 mg/kg body weight/day) via intraperitoneal injection (i.p.) every day, another group received both vitamin C in the water supply and buparlisib via i.p. For both experiments, the control group received vehicle only. Tumor volume and body weight were measured by caliper and scale. After extraction, tumors were washed in PBS, weighed, and then fixed for further experiments. Organs (liver and lungs) were obtained for analyzing metastasis.

### RNA-seq

BT20 cells cultured in 6-well plates were treated with or without vitamin C (100 μM) for 5 days. The medium was changed daily before each treatment to avoid the accumulation of vitamin C. Total RNA was then extracted from the cells using the RNeasy Mini Kit (Qiagen). Whole transcriptome sequencing (RNA-seq) was carried out at the Sequencing Core Facility of the John P. Hussman Institute of Human Genomics at the University of Miami. Statistical significance was determined using 2 different differential expression calculators (edgeR and DESeq2), as conducted in our previous studies 22. To reduce false positives, only genes with an adjusted *P*-value below 0.05 across both methods were considered differential. Pathway and functional enrichment analysis of RNA‐seq data was done using Enrichr and GSEA. Annotations were ranked by combined *P* value and *Z* scores.

### Immunofluorescence and image analysis

BT20 and MDA-MB-453 cells were seeded on coverslips for 24 hours followed by treatment with vitamin C (100 μM) in combination with buparlisib (0.5 μM). After the treatment, cells were washed with cold PBS and fixed for 10 minutes at room temperature with 4% paraformaldehyde, permeabilized for 5 minutes with 0.2% Triton X-100 PBS and blocked for 30 minutes with 5% BSA. This was followed by incubation with anti-KDM5A antibody (1:500) (Cell Signaling, Danvers MA), overnight at 4°C, PBS wash, and then by the secondary antibodies at 1:1000 dilution for another hour at room temperature. To stain the nucleus, cells were incubated with 40 μg/ml DAPI for 20 minutes at room temperature. All fluorescence images were acquired using a Zeiss LSM 710 confocal microscope and captured into a 512 x 512 frame size by averaging 4 times at a bit depth of 16. Fluorescence intensities from the images were quantified using ImageJ. Average intensity values were measured from every cell within the image field from a minimum of three 20× images per condition. The intensity values from individual cells were plotted and statistically analyzed by one-way *ANOVA* with a Tukey post hoc test using GraphPad Prism 7 (GraphPad Software, San Diego, CA).

### Western blot

Total proteins extracted from BT20 and MDA-MB-453 cells were loaded onto 4-15% gradient polyacrylamide gels and then transferred to PVDF membrane (Bio-Rad, Hercules, CA). After being blocked by 5% non-fat milk for 1 h at room temperature, the membrane was incubated in primary antibodies. Primary antibodies used in this study include anti-AKT1, anti-AKT2, anti-mTOR, anti-GSK3, anti-H3K4me3, anti-PCNA, anti-AUKB, anti-fascin, (Cell signaling, Denver, MA) and anti-FILIP1L (Abcam, Cambridge, UK). Proteins were visualized by chemiluminescence using ECL substrate (Thermo Fisher Scientific, Carlsbad, CA). To ensure equal loading, the membrane was stripped and reprobed by mouse anti-H3 monoclonal antibody or anti-CYBP antibody (1:1,000, Santa Cruz Biotechnology, Dallas, TX). The densities of the bands were captured by ImageJ. Statistical significance of differences in band density among different treatment groups were assessed by *ANOVA*. To test if the gene expression by vitamin C is dependent on TET-mediated DNA demethylation, TETs were silenced by siRNA. Individual Accell siRNA targeted against human *TET1, TET2,* and *TET3* were designed and synthesized by Dharmacon (Lafayette, CO) and transfected following the manufacturer's instruction. BT20 cells were plated and grown until achieving 30-50% confluence. Transfection of siRNA was performed using Lipofectamine 2000 (Thermo Fisher). Media was changed after 6 hours of transfection to eliminate the possible toxicity of transfecting reagents. Cells were harvested after 5 days for protein extraction and subsequent Western blot.

### Statistical analysis

All observations in this study were analyzed in triplicate or greater and each experiment was repeated three times. GraphPad Prism was also used to generate and analyze data. Dose response data were analyzed by *ANOVA* followed by a Tukey Post hoc comparison. Values represent the mean ± SEM of three independent experiments. To compare two groups, student's *t*-test was used and *P* < 0.05 was considered as statistically significant.

## Results

### Vitamin C improves the efficacy of buparlisib in treating TNBC cells

**Figure 1:**
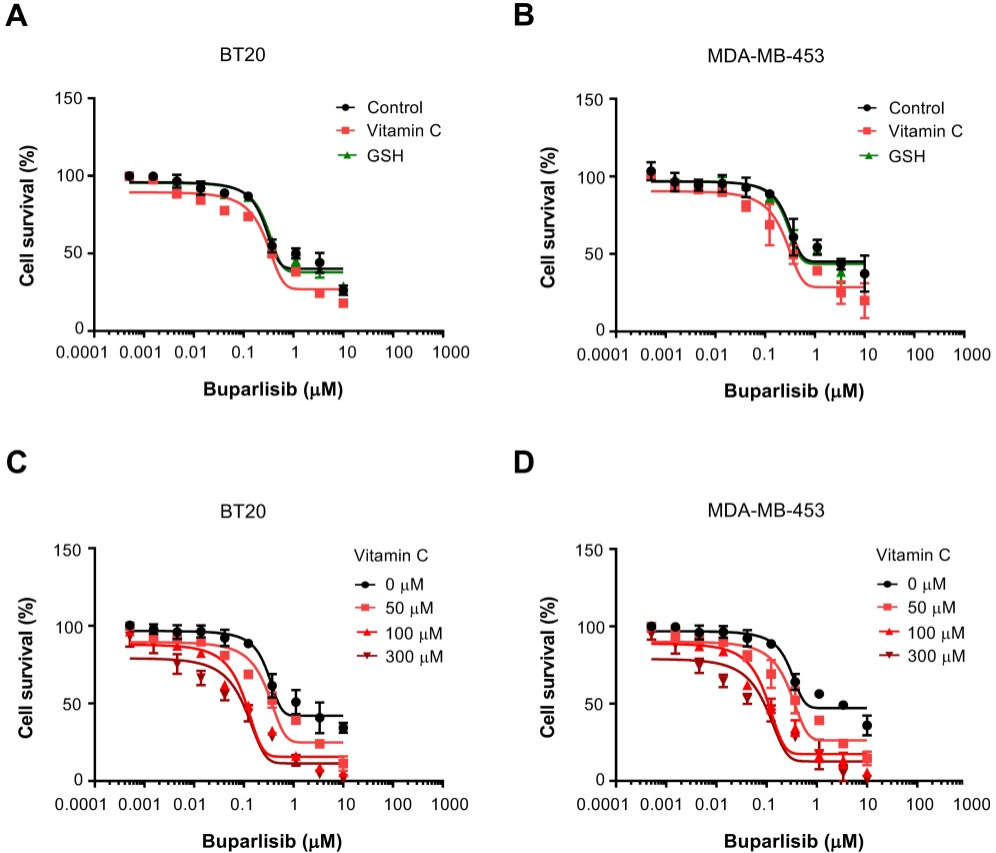
** Vitamin C increases the efficacy of buparlisib. (A)** Vitamin C (50 μM) decreases the EC_50_ of buparlisib in BT20 cells. **(B)** Vitamin C (50 μM) decreases the EC_50_ of buparlisib in MDA-MB-453 cells. GSH has no obvious effect on the EC_50_ of buparlisib. **(C)** Both 100 and 300 μM vitamin C decrease the EC_50_ of buparlisib further by ~3.5-fold compared to 50 μM vitamin C in BT20 cells. **(D)** Both 100 and 300 μM vitamin C decrease the EC_50_ of buparlisib further by ~3.5-fold compared to 50 μM vitamin C in MDA-MB-453 cells.

The average vitamin C level in the plasma is ~50 μM in healthy humans 24. We first tested if vitamin C at 50 μM has any impact on the efficacy of PI3K inhibitor buparlisib in treating TNBC cells including BT20 and MDA-MB-453, which carry activating *PIK3CA* mutations. BT20 cells have two compound mutations (P539R, H1047R) and MDA-MB-453 cells carry one mutation (H1047R) in *PIK3CA*. Without vitamin C in the media, the EC_50_ of buparlisib was 1.4 μM for BT20 cells and 1.8 μM for MDA-MB-453 cells. After addition of 50 μM vitamin C, the EC_50_ of buparlisib was reduced to 0.47 μM for BT20 cells and 0.46 μM for MDA-MB-453 cells, about a 3-fold reduction. In contrast, glutathione (GSH) had no obvious effect (Figure 1A-B). GSH is known as a general antioxidant and can quickly enter into cells through GSH transporters and connexin hemichannels 25, 26. Despite its potent antioxidant action, GSH does not alter the response of TNBC cells to buparlisib while vitamin C does, suggesting that the antioxidant effect of vitamin C alone is not mediating its action on PI3K inhibitor.

By diet and oral delivery, plasma vitamin C level can reach 100~200 μM 24. Concentrations greater than 300 μM of vitamin C in the plasma would require intravenous infusion or intraperitoneal injection. We then tested if higher vitamin C concentrations could further change the EC_50_ of buparlisib compared to vitamin C at 50 μM. With the addition of 100 μM vitamin C, the EC_50_ of buparlisib was further decreased to 0.12 μM for BT20 cells and 0.11 μM for MDA-MB-453 cells, nearly a 4-fold reduction compared to vitamin C at 50 μM (Figure 1C-D). Vitamin C at 300 μM further reduced the EC_50_ of buparlisib to 0.066 μM for BT20 cells and 0.055 μM for MDA-MB-453 cells, indicating that the effect of vitamin C at 300 μM is moderately better than at 100 μM. Overall, these results suggest that vitamin C, especially at 100 μM which could be achieved conveniently by oral delivery, significantly increases the efficacy of buparlisib in treating TNBC cells.

### Vitamin C improves the response of TNBC to buparlisib *in vitro*

**Figure 2:**
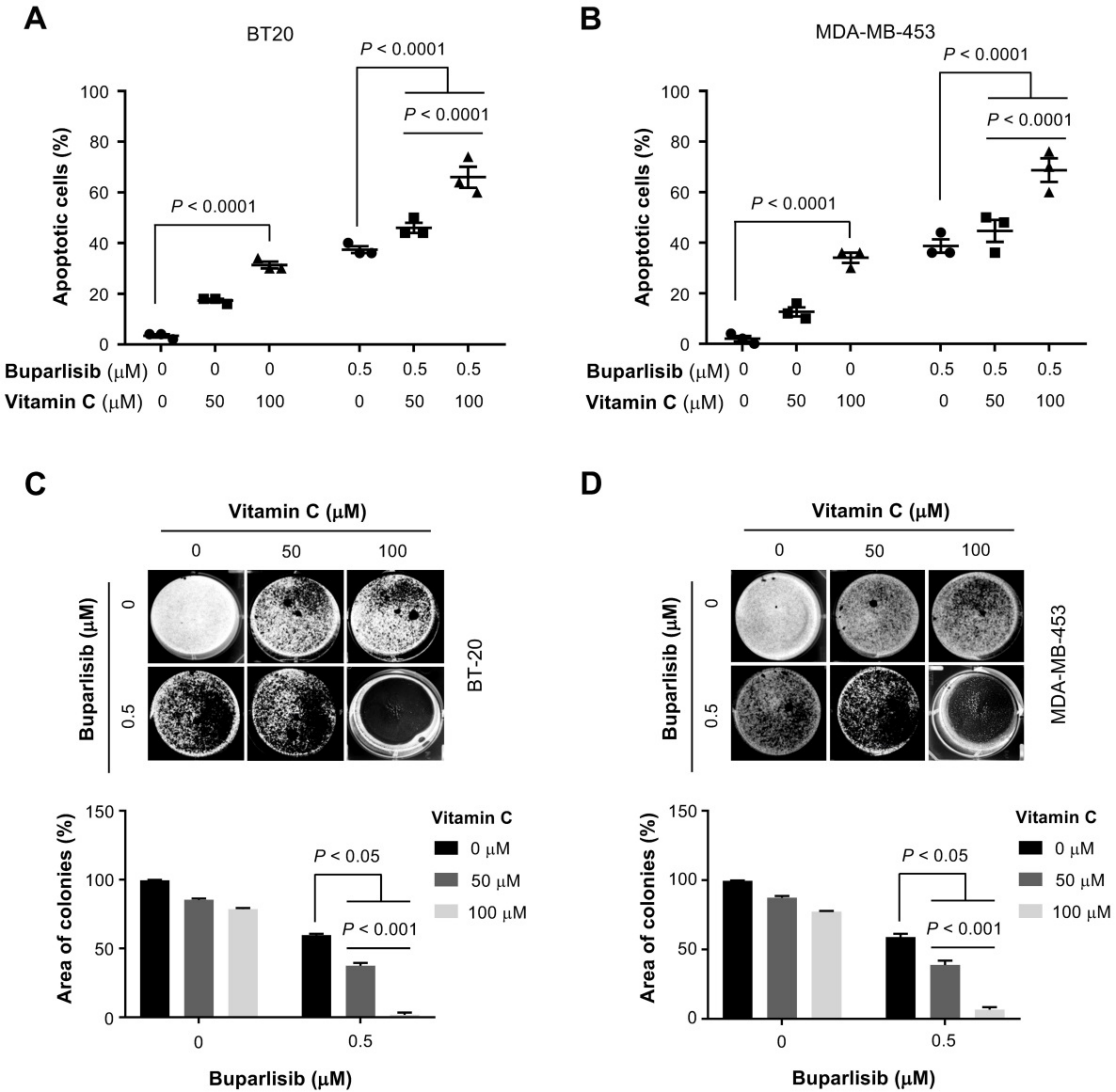
** Vitamin C improves the response of TNBC cells to buparlisib. (A)** Co-treatment with vitamin C (100 μM) and buparlisib (0.5 μM) induces more apoptosis than co-treatment with buparlisib and 50 μM vitamin C or buparlisib alone (*P* < 0.0001) in BT20 cells. **(B)** Co-treatment with vitamin C (100 μM) and buparlisib (0.5 μM) induces more apoptosis than co-treatment with buparlisib and 50 μM vitamin C or buparlisib alone (*P* < 0.0001) in MDA-MB-453 cells. **(C)** Co-treatment with vitamin C (100 μM) and buparlisib (0.5 μM) almost completely blocks colony formed by BT20 cells compared to co-treatment with buparlisib and 50 μM vitamin C or buparlisib alone (*P* < 0.001). **(D)** Co-treatment with vitamin C (100 μM) and buparlisib (0.5 μM) almost completely blocks colony formed by MDA-MB-453 cells compared to co-treatment with buparlisib and 50 μM vitamin C or buparlisib alone (*P* < 0.001).

Given the enhanced efficacy by vitamin C on cell viability *in vitro*, we next tested the co-treatment of cultured TNBC cells with buparlisib and vitamin C. An apoptosis assay was used to evaluate cell death in order to validate the initial EC_50_ findings. The result showed vitamin C (50 μM) alone did not induce apoptosis, while vitamin C at 100 μM slightly increased apoptotic cells. Buparlisib (0.5 μM) promoted apoptosis. In combination, vitamin C enhanced the effect of buparlisib to induce apoptosis in BT20 and MDA-MB-453 cells (Figure 2A-B). Co-treatment of TNBC cells with 100 μM vitamin C and buparlisib further induced apoptosis compared to co-treatment with 50 μM vitamin C and buparlisib or buparlisib alone.

We then examined vitamin C and buparlisib co-treatment on TNBC colony formation. Buparlisib (0.5 μM) inhibited TNBC colony formation while vitamin C alone had no obvious effect. Combined treatment with buparlisib and vitamin C further decreased colony formation (Figure 2C-D). Comparatively, 100 μM vitamin C with buparlisib more effectively inhibited colony formation compared to co-treatment with 50 μM vitamin C and buparlisib or buparlisib alone. Collectively, these results indicate that vitamin C at 100 μM, which is achievable in the plasma *in vivo* by oral delivery, significantly enhances the effect of buparlisib to suppress TNBC malignancy *in vitro*.

### Vitamin C improves the response of TNBC to buparlisib *in vivo*

Humans as well as other high primates can no longer synthesize vitamin C due to a mutated and nonfunctional L-gulonolactone oxidase (Gulo), the enzyme catalyzing one key step of vitamin C biosynthesis. For humans, vitamin C must be supplied in the diet or via oral supplements. In contrast, experimental rodents such as mice synthesize vitamin C *de novo* in the liver. We examined TNBC xenograft in immune-deficient NSG mice, in which endogenous vitamin C concentration in the plasma is around 50 μM, similar to the level observed in healthy humans. Our cell-based experiments showed that 100 μM vitamin C treatment is superior to 50 μM, for enhancing the anti-cancer effect of buparlisib. To reach 100~200 μM vitamin C in the plasma, additional vitamin C (3.3 g/L) was provided in the drinking water of NSG mice, as previously described 27.

**Figure 3:**
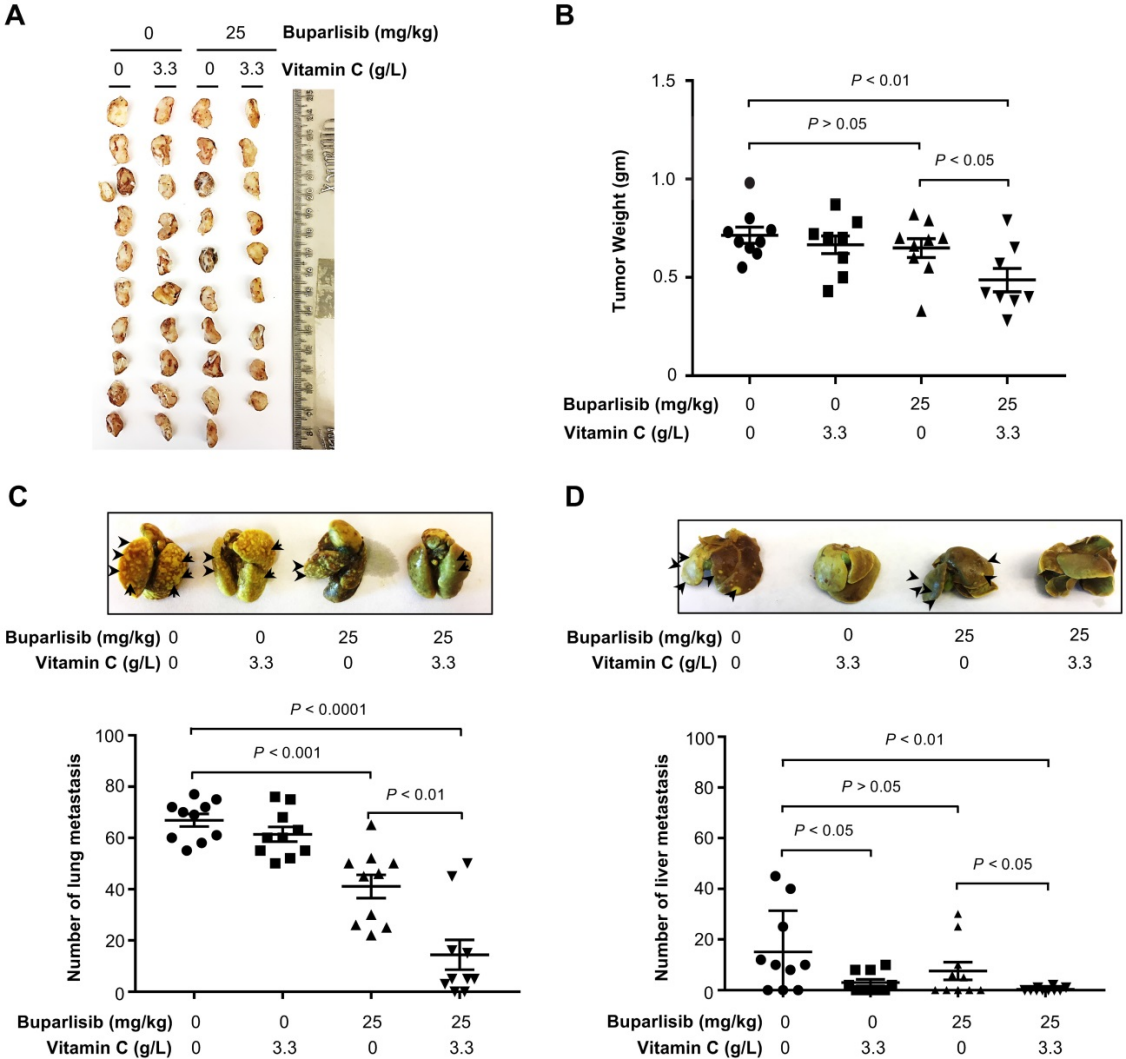
** Vitamin C improves the response of TNBC xenografts to buparlisib treatment. (A)** Photograph of human BT20 TNBC xenografts from female NSG mice treated with or without buparlisib (25 mg/kg body weight) and supplemented with or without vitamin C (3.3 g/L) in the drinking water. **(B)** Quantification of xenograft weights shows that xenografts in buparlisib alone group are similar to non-treated group (*P* > 0.05). Xenografts in buparlisib and vitamin C co-treatment group are smaller compared to non-treated group (*P* < 0.01) or compared to buparlisib alone group (*P* < 0.05). **(C)** Representative lung images and quantification show metastatic nodules in buparlisib alone group are less compared to non-treated group (*P* < 0.001). Metastatic nodules are less in buparlisib and vitamin C co-treatment group compared to non-treated group (*P* < 0.0001) or compared to buparlisib alone group (*P* < 0.01). **(D)** Representative liver images and quantification show metastatic nodules in buparlisib alone group are similar to non-treated group (*P* > 0.05). Metastatic nodules in vitamin C alone group are less compared to non-treated group (*P* < 0.05). Co-treatment with buparlisib and vitamin C further reduces metastatic nodules in the liver compared to non-treated group (*P* < 0.01) or compared to buparlisib alone group (*P* < 0.05).

We then tested the impact of vitamin C and a dose of buparlisib that should be sub-therapeutic in xenograft models, as shown previously 28. Buparlisib (25 mg/kg body weight by i.p. daily for 2 weeks) was administrated and vitamin C (3.3 g/L) was provided in the drinking water. We chose to deliver buparlisib by i.p. in order to avoid any potential direct interaction of the two agents in the digestion system. Vitamin C (3.3 g/L) showed no obvious tumoristatic effect when administered alone (*P* > 0.05). Buparlisib at this sub-therapeutic dose did not inhibit xenograft growth (*P* > 0.05) either. Only buparlisib in combination with vitamin C measurably attenuated BT20 xenograft growth compared to vehicle controls (*P* < 0.01) or compared to buparlisib alone group (*P* < 0.05) (Figure 3A-B, S1A).

The improved efficacy of buparlisib by vitamin C was further revealed as a reduction in tumor metastasis to other organs. Buparlisib at the sub-therapeutic dose alone reduced formation of lung metastasis (*P* < 0.001). The combination of buparlisib and vitamin C further decreased metastatic lung nodules compared to vehicle controls (*P* < 0.0001) or compared to buparlisib alone group (*P* < 0.01) (Figure 3C). Distinct from the lung, metastasis to the liver was attenuated by vitamin C alone (*P* < 0.05), but not by buparlisib alone (*P* > 0.05). Co-treatment with buparlisib and vitamin C caused a greater decrease in liver metastasis compared to vehicle group (*P* < 0.01), or compared to buparlisib alone group (*P* < 0.05) (Figure 3D). Taken together, these results suggest that vitamin C sensitizes TNBC to PI3K inhibition treatment, rendering a buparlisib dose that is sub-therapeutic when given alone, capable of significantly inhibiting TNBC xenograft growth and metastasis.

### Vitamin C and buparlisib cooperatively promote H3K4 demethylation

Classic PI3K signaling, via AKT and mTOR, controls a variety of essential cellular processes. It has recently been revealed that aberrant PI3K signaling also affects histone methylation, which further contributes to cancer progression 29, 30. In *PIK3CA*^H1047R^-mutated breast cancer, the phosphorylation of KDM5A by the overactivated AKT blocks its translocation to the nucleus, which limits the access of KDM5A to its substrates, H3K4me2 and H3K4me3. This results in high H3K4me3, which is a poor prognostic epigenetic mark for cancer 31. AKT inhibitors increase the presence of KDM5A in the nucleus by blocking its phosphorylation, which leads to a reduction of H3K4me3 and further suppresses the transcription of genes associated with poor clinical outcome 30.

Vitamin C is known to promote the enzymatic activity of JmjC domain-containing histone demethylases, which has, however, only been demonstrated in stem cells 19, 20. KDM5A is one member of the JmjC domain-containing histone demethylase family. We reasoned that vitamin C, in cooperation with PI3K inhibition, could decrease H3K4me3 in TNBC cells by facilitating KDM5A-mediated demethylation. To test this hypothesis, we first examined KDM5A nuclear translocation. Buparlisib increased the presence of KDM5A in the nucleus of the BT20 cell (Figure S2A), as did AKT inhibitor MK2206 30. Treatment of BT20 cells with vitamin C (100 μM) also slightly increased KDM5A nuclear translocation. Co-treatment with buparlisib and vitamin C further increased the level of KDM5A in the nuclei of BT20. The enhanced nuclear translocation of KDM5A by buparlisib was validated by Western blot of cellular fractions of nucleus and cytoplasm (Figure S2B).

**Figure 4:**
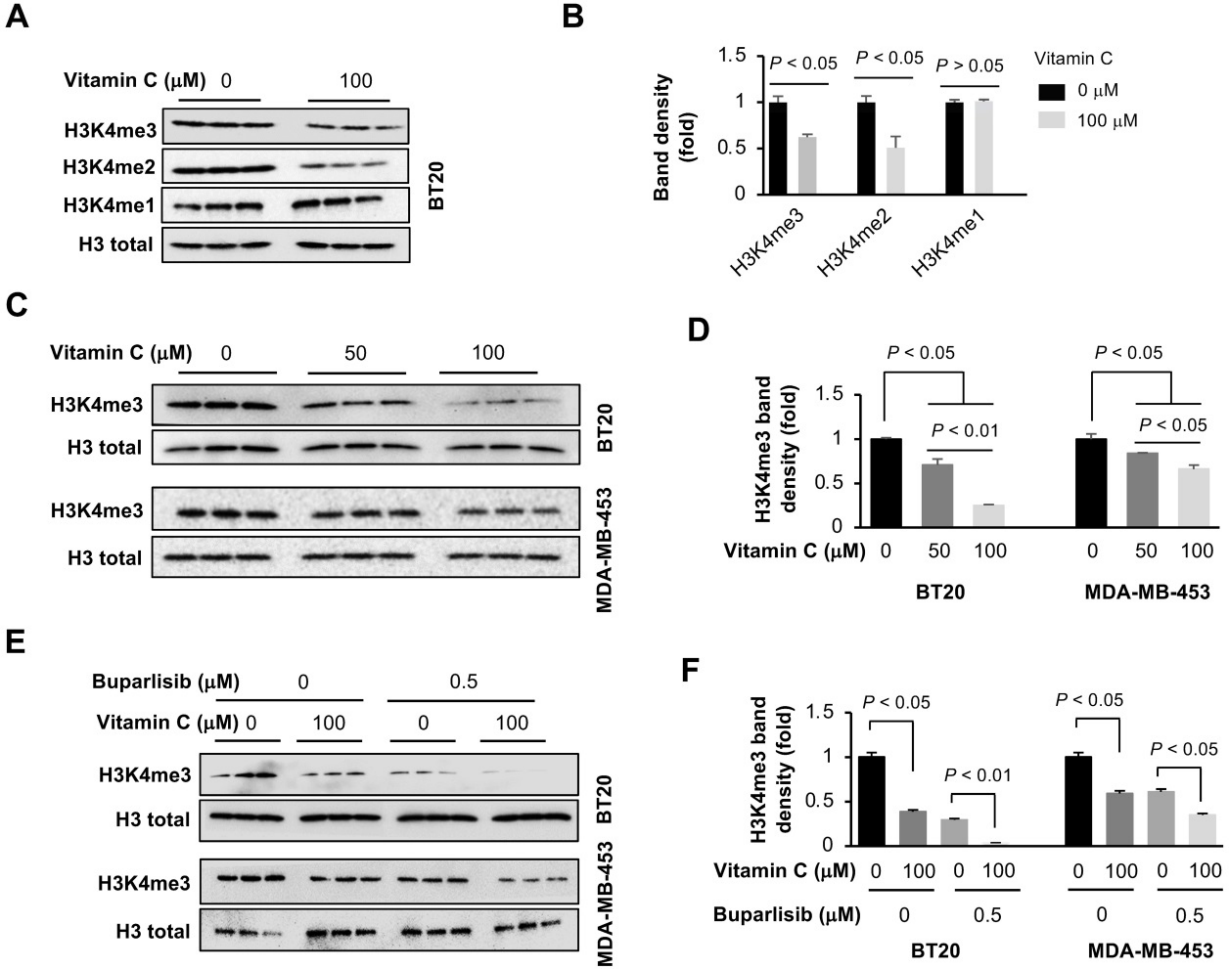
** Vitamin C and buparlisib cooperatively reduce H3K4 methylation. (A)** Western blot of H3K4 methylation marks in BT20 cells treated with vitamin C. **(B)** Semi-quantification of band density shows that vitamin C (100 μM) reduces H3K4me3 and H3K4me2, but not H3K4me1, in BT20 cells. **(C)** Western blot of H3K4me3 in BT20 and MDA-MB-453 cells treated with different concentrations of vitamin C. **(D)** Semi-quantification of band density shows that compared to 50 μM, vitamin C at 100 μM further decreases H3K4me3 in BT20 and MDA-MB-453 cells. **(E)** Western blot of H3K4me3 in BT20 and MDA-MB-453 cells treated with vitamin C and buparlisib. **(F)** Semi-quantification of band density shows that vitamin C (100 μM) alone or buparlisib (0.5 μM) alone reduces H3K4me3 in BT20 and MDA-MB-453 cells. Co-treatment with vitamin C and buparlisib further reduces H3K4me3 in these TNBC cells.

Once in the nucleus, KDM5A preferentially demethylates H3K4, an activity that could be promoted by vitamin C. We evaluated the impact of vitamin C and buparlisib on H3K4 methylation in TNBC cells by Western blot. Vitamin C (100 μM) alone decreased H3K4me3 and H3K4me2, but not H3K4me1, in BT20 cells (Figure 4A-B), which could be explained by the preferential demethylation of H3K4me2 and H3K4me3 by KDM5A. Compared to 50 μM, vitamin C at 100 μM further reduced H3K4me3, both in BT20 and MDA-MB-453 cells (Figure 4C-D). In contrast, other histone trimethylation marks, such as H3K9me3, H3K27me3 and H3K36me3, remained largely unchanged in BT20 cells after vitamin C treatment (Figure S3). Furthermore, buparlisib (0.5 μM) alone also decreased H3K4me3 in BT20 and MDA-MB-453 cells. Co-treatment of vitamin C and buparlisib further suppressed H3K4me3 (Figure 4E-F). Taken together, these results indicate that buparlisib and vitamin C cooperatively decrease H3K4me2 and H3K4me3 by increasing the presence of KDM5A in the nucleus and by serving as a cofactor to promote KDM5A-mediated H3K4 demethylation.

### Vitamin C and buparlisib cooperatively block PI3K signaling

**Figure 5:**
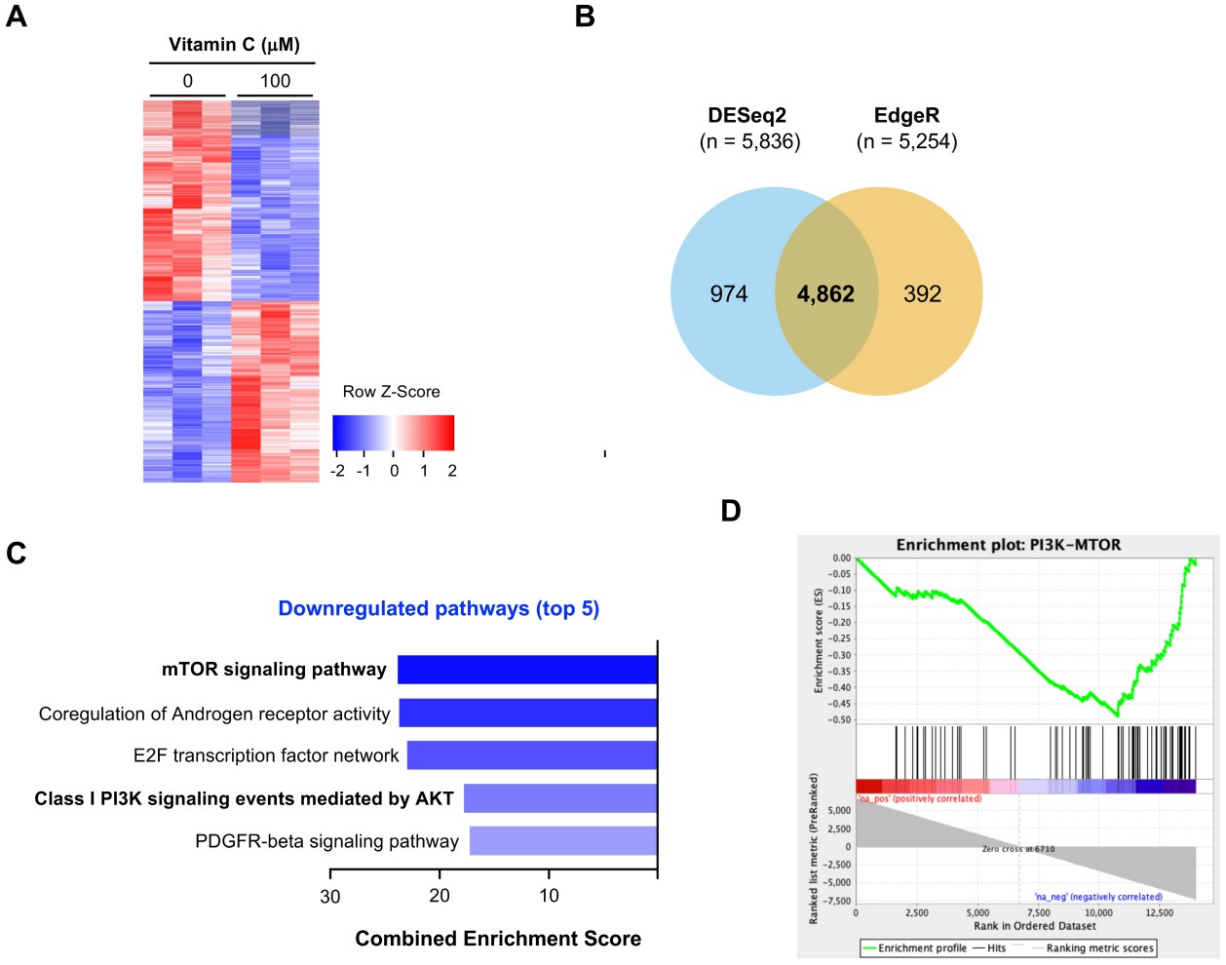
** The impact of vitamin C on the transcriptome of BT20 cells. (A)** Vitamin C (100 μM) changes the transcriptome of BT20 cells as shown by heatmap. **(B)** A total of 4,862 transcripts is changed by vitamin C treatment, which are called by both DESeq2 and edgeR. **(C)** Two of the top 5 downregulated pathways can be merged as one PI3K-mTOR pathway. **(D)** Vitamin C changes the expression of genes in the PI3K pathway, which predicts a downregulation of PI3K-mTOR signaling as shown by pathway enrichment analysis.

H3K4 methylation, especially H3K4me2 and H3K4me3 in promoters, is closely associated with gene transcription 32. Decreased H3K4me2 and H3K4me3 after vitamin C treatment would result in a change in gene transcriptions. We employed RNA-seq to assess the impact of vitamin C on the whole transcriptome. BT20 cells were treated with or without vitamin C (100 μM) for 5 days. Total RNAs were extracted and submitted for RNA-seq. We observed a significant shift in the BT20 transcriptome after vitamin C treatment, as shown by the heatmap (Figure 5A). A total of 5,836 genes were determined to be differentially expressed by DESeq2, and 5,254 genes were determined to be differentially expressed by edgeR. Of these, 4,862 genes were significantly and differentially expressed using both methods (Figure 5B). These results suggest that the transcriptome of BT20 cells is significantly changed by vitamin C treatment.

We conducted further analyses to identify which pathways are affected by vitamin C. The result of NCI-Nature Pathway Interaction analyses showed that no pathway is significantly upregulated by vitamin C in BT20 cells. In contrast, multiple pathways were downregulated by vitamin C, prominently the PI3K pathway shown as an mTOR signaling pathway and class I PI3K signaling events mediated by AKT (Figure 5C). According to the analyses, the transcription of multiple genes in PI3K pathway, including AKT2, mTOR, GSK3α and mLST8, was changed by vitamin C. The transcription of these genes in the PI3K pathway was suppressed by vitamin C as shown in a GSEA enrichment plot (Figure 5D).

**Figure 6:**
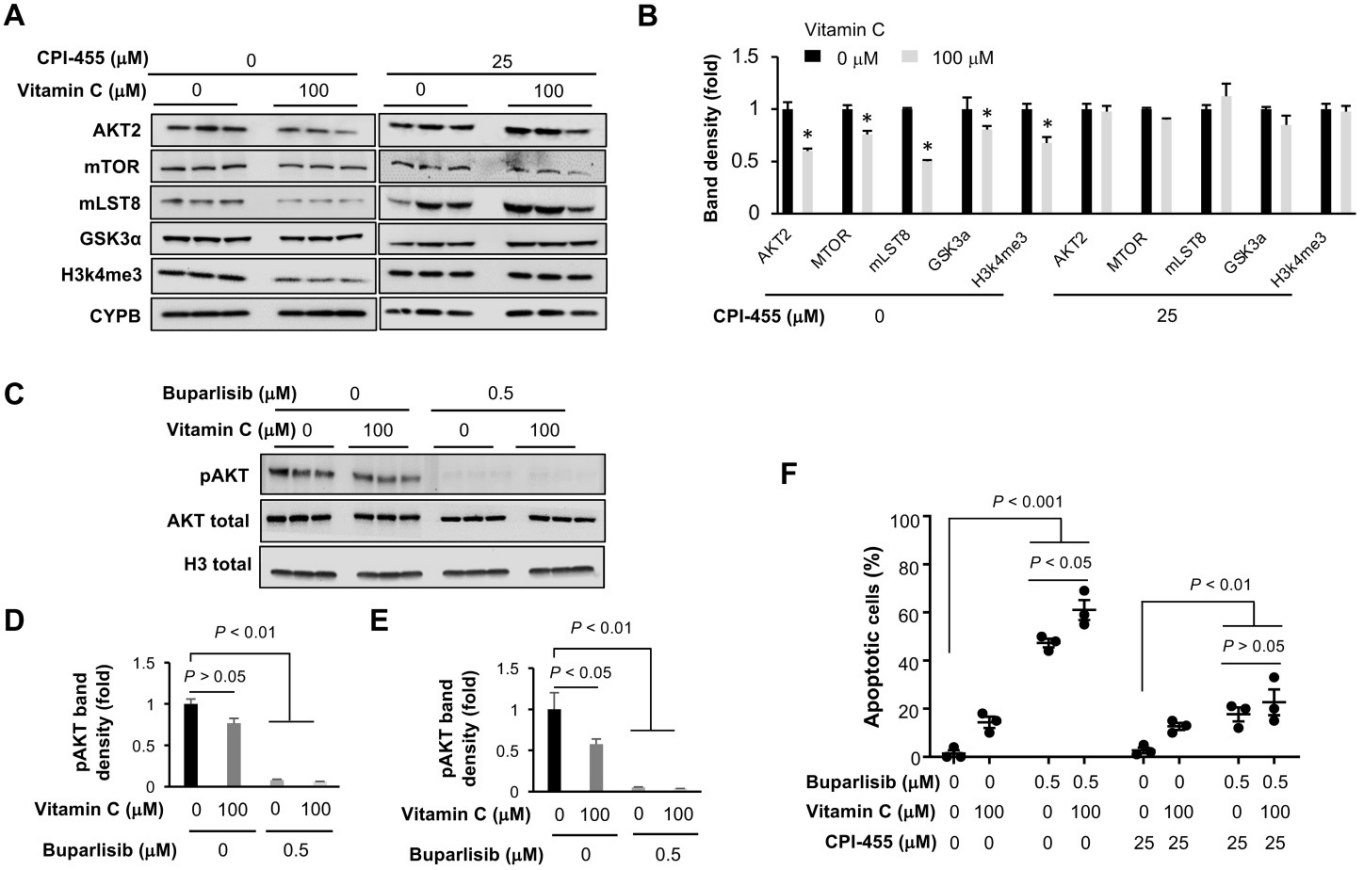
** Vitamin C improves the response of TNBC cells to buparlisib in a KDM5 dependent manner. (A)** Western blot of H3K4me3 and PI3K pathway genes in BT20 cells treated with vitamin C and KDM5 inhibitor CPI-455. **(B)** Semi-quantification of band density shows that vitamin C (100 μM) decreases the protein level of AKT2, mTOR, GSK3α, mLST8 as well as H3K4me3 in BT20 cells. This effect of vitamin C on H3K4me3 and the PI3K pathway proteins is largely abolished by CPI-455 (25 μM). **(C)** Western blot of phosphorylated AKT (Ser473, pAKT) in BT20 cells treated with vitamin C and buparlisib. **(D)** Semi-quantification of band density shows that buparlisib indeed inhibits pAKT, while vitamin C alone only marginally reduced pAKT (*P* = 0.056), when normalized with total AKT. **(E)** When normalized with histone H3 since vitamin C decreases AKT2 expression, vitamin C alone also reduces pAKT (*P* < 0.05). Co-treatment with buparlisib and vitamin C almost completely diminished pAKT. **(F)** CPI-455 largely abolishes the promotion of buparlisib-induced apoptosis by vitamin C in BT20 cells.

We then used Western blotting to verify the downregulation of key genes in the PI3K pathway, especially AKT and direct AKT substrates or binding partners. The inhibited expression of AKT2, mTOR, GSK3α and mLST8 (target of rapamycin complex subunit 8−part of mTOR complex) by vitamin C was subsequently validated at the protein level (Figure 6A-B). Housekeeper protein cyclophilin B was used as a loading control since the transcription of housekeeping genes GPDH and β-actin was changed in BT20 cells by vitamin C treatment, as shown in our RNA-seq data.

To test if vitamin C instigated suppression of these PI3K pathway genes is dependent on TET-mediated DNA demethylation, the expression of TETs was knocked down in BT20 cells by siRNA. Vitamin C (100 μM) continued to inhibit the expression of these PI3K pathway genes after the knockdown of TETs (Figure S4), suggesting that vitamin C downregulates the expression of genes in the PI3K pathway, likely in a TETs independent manner. CPI-455, an inhibitor of H3K4 demethylase KDM5, was then applied to test if vitamin C changed the expression of PI3K pathway genes dependent on KDM5-mediated H3K4 demethylation. CPI-455 largely abolished the reduction of H3K4me3 induced by vitamin C, indicating that vitamin C decreases H3K4me3 likely by promoting KDM5-mediated demethylation. Importantly, vitamin C no longer inhibited the expression of AKT2, mTOR, GSK3α, and mLST8 after CPI-455 pretreatment (Figure 6A-B). The effects of CPI-455 were further validated by knocking down the expression of KDM5 (Figure S5). These results suggest that vitamin C, via KDM5-mediated H3K4 demethylation, suppresses the expression of genes in the PI3K pathway.

Next, we assessed the impact of buparlisib and vitamin C co-treatment on PI3K signaling using phosphorylation of AKT (pAKT) at serine 473 as a readout. Buparlisib alone inhibited pAKT in BT20 cells, while vitamin C alone only marginally reduced pAKT when normalized with total AKT (*P* = 0.056; (Figure 6C-D). However, when normalized with housekeeper proteins such as histone H3 due to the altered AKT expression by vitamin C, vitamin C treatment alone also reduced the level of pAKT in BT20 cells (Figure 6E). Vitamin C and buparlisib co-treatment almost completely diminished pAKT. Overall, these data suggest that vitamin C and buparlisib could coordinately block PI3K signaling by decreasing PI3K downstream proteins, and by inhibiting PI3K enzymatic activity.

As described above, the altered expression of PI3K pathway genes by vitamin C was dependent on KDM5, raising a possibility that KDM5 may mediate the effect of vitamin C on the response of TNBC to buparlisib. We found that KDM5 inhibitor CPI-455 markedly inhibited apoptosis induced by buparlisib with or without vitamin C co-treatment. More importantly, CPI-455 eliminated the differences in apoptosis induction between the vitamin C + buparlisib group and the buparlisib alone group (Figure 6F). These results suggest that KDM5 likely mediates, at least partially, the effect of vitamin C on the improved response of TNBC to PI3K inhibition.

### Vitamin C and buparlisib cooperatively regulate the expression of genes critical to cancer growth and metastasis

Previously, decreased H3K4me3 by AKT inhibitor MK2206 in *PIK3CA*-mutated TNBC cells was shown to be associated with the suppressed expression of aurora kinase B (AURKB), proliferating cell nuclear antigen (PCNA) and other genes that are critical to breast cancer survival 30. We evaluated the impact of vitamin C and buparlisib on AURKB and PCNA in TNBC cells. The results showed that vitamin C (100 μM) alone or buparlisib (0.5 μM) alone decreased both AURKB and PCNA in BT20 and MDA-MB-453 cells. Co-treatment of vitamin C and buparlisib further suppressed AURKB and PCNA (Figure S6). These results suggest that vitamin C and buparlisib cooperatively suppress the expression of genes critical to tumor growth.

**Figure 7:**
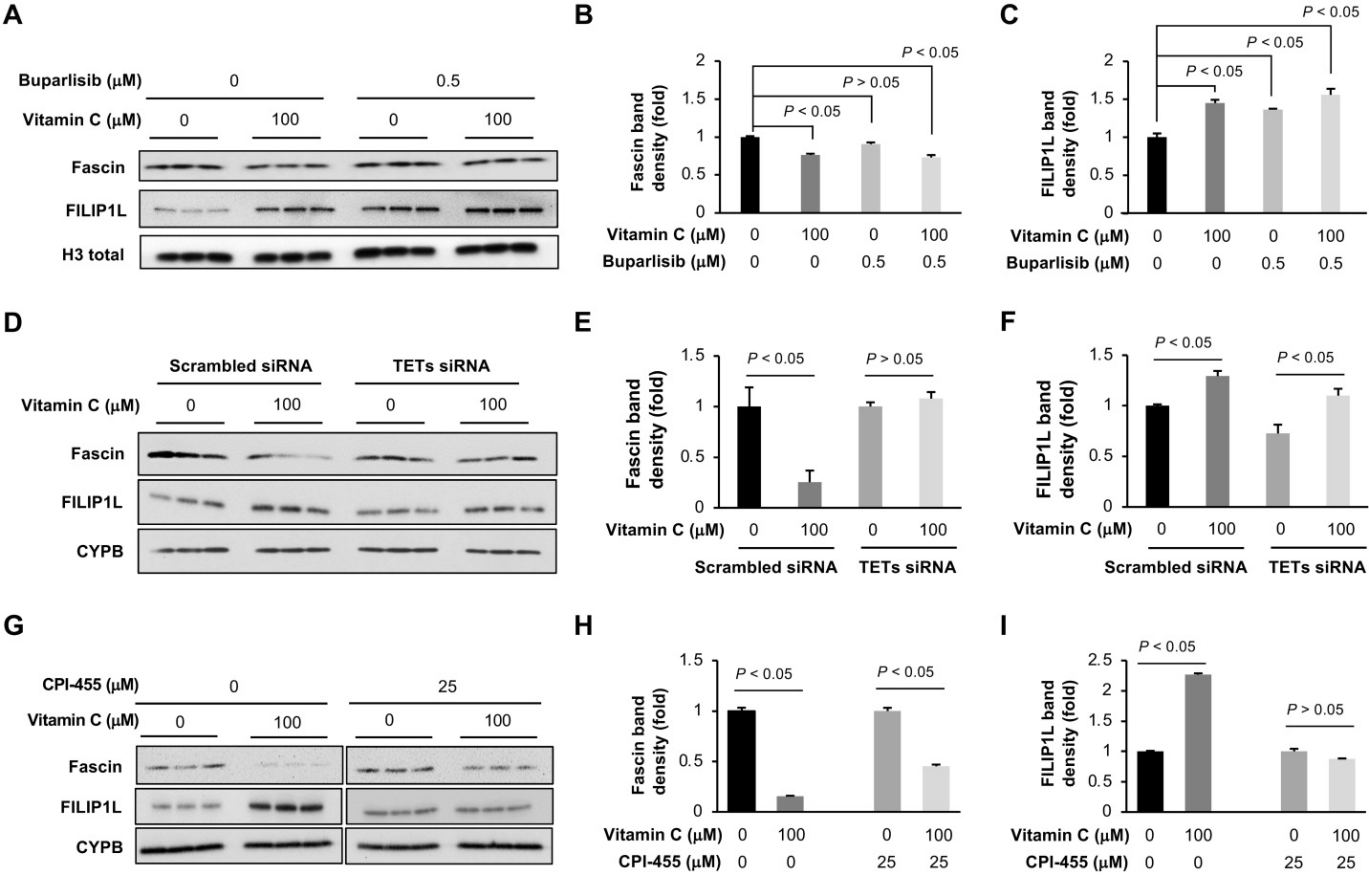
** Vitamin C and buparlisib cooperatively decrease the expression of genes critical to cancer metastasis. (A)** Western blot of fascin and FILIP1L in BT20 cells treated with vitamin C and buparlisib. **(B)** Semi-quantification of band density shows that vitamin C (100 μM) alone reduces fascin while buparlisib (0.5 μM) alone has no effect on fascin. **(C)** Semi-quantification of band density shows that vitamin C (100 μM) alone, or buparlisib (0.5 μM) alone, increases FILIP1L in BT20 cells. **(D)** Western blot of fascin and FILIP1L in BT20 cells treated with TETs siRNA followed by vitamin C. **(E)** Semi-quantification of band density shows that TETs siRNA abolishes the downregulation of fascin by vitamin C. **(F)** Semi-quantification of band density shows that TETs siRNA does not affect the upregulation of FILIP1L by vitamin C. **(G)** Western blot of fascin and FILIP1L in BT20 cells treated with vitamin C and CPI-455. **(H)** Semi-quantification of band density shows that CPI-455 does not affect the downregulation of fascin by vitamin C. **(I)** Semi-quantification of band density shows that CPI-455 abolishes the upregulation of FILIP1L by vitamin C.

The improved efficacy of buparlisib by vitamin C was more profoundly demonstrated at cooperative inhibition of tumor metastasis to other organs. To understand how vitamin C and buparlisib together inhibit TNBC metastasis, we examined the transcription of genes known to be involved in metastasis 33 in our RNA-seq data. We discovered that the transcription of 36 metastasis-related genes was changed by vitamin C treatment (Table S1). The most downregulated gene is fascin (0.5-fold), which is known to increase metastasis 34. The most upregulated gene is filamin A interacting protein 1-like (FILIP1L) (3.1-fold), which inhibits cancer metastasis when overexpressed 35. The decreased fascin and increased FILIP1L in BT20 cells by vitamin C were further validated by Western blot (Figure 7A-C) and in xenograft by immunohistochemistry (Figure S1B-E). Buparlisib alone also increased FILIP1L but had no obvious effect on fascin. Buparlisib and vitamin C co-treatment decreased fascin and elevated FILIP1L. Knockdown of TETs by siRNA abolished the downregulation of fascin but not the upregulation of FILIP1L by vitamin C, while KDM5 inhibitor CPI-455 abolished the upregulation of FILIP1L, but not the downregulation of fascin by vitamin C (Figure 7D-I), indicating that KDM5 is likely responsible for the upregulation of FILIP1L and TETs largely mediate the downregulation of fascin by vitamin C. Overall, these results suggest that vitamin C improves the anti-tumor activity of buparlisib by cooperatively changing the expression of genes critical to TNBC growth and metastasis.

## Discussion

Lack of targeted therapy is a major barrier for the clinical care of patients with TNBC. PI3K inhibition has been considered a promising targeted therapy for TNBC due to frequently mutated *PIK3CA* and other genes in the pathway. PI3K inhibitors, including pan PI3K inhibitor buparlisib and PI3Kα specific inhibitors, such as alpelisib and taselisib, are under investigation in ongoing clinical trials for TNBC treatment. However, completed randomized and double-blinded clinical trials, mainly for other breast cancer subtypes, have shown somewhat disappointing outcomes, with a mild improvement of progression-free survival and relatively severe toxicities 10-13. The low response of breast cancer to PI3K inhibitors and the toxicities jeopardize the prospect of subsequent clinical trials and the ultimate application of these drugs in patient care.

This current preclinical study provides evidence for a potentially practical solution to improve the response of TNBC to buparlisib, thereby expanding the therapeutic window of buparlisib. We show that vitamin C improves the efficacy of buparlisib in treating *PIK3CA*-mutated TNBC cells *in vitro*. Vitamin C at 100 μM, a dose achievable *in vivo* by oral delivery, is more potent than 50 μM, and promotes the effect of buparlisib to induce apoptosis and to inhibit survival and colony formation. In contrast, vitamin C at 300 μM, a dose that requires intravenous infusion or intraperitoneal injection, does not dramatically further enhance buparlisib efficacy. With oral vitamin C administration, a buparlisib dose that had no obvious anti-tumor activity on its own was shown to inhibit growth of both primary and metastatic xenografts. In both liver and lung lesions, the anti-metastatic effect of dual therapy exceeded that of either agent alone. Notably, no obvious toxicities, including body weight loss, were observed. Future studies will determine if vitamin C can also improve the response of TNBC to other PI3K inhibitors, especially alpelisib, which was recently approved by the FDA to treat *PIK3CA*-mutated HR+/HER2- breast cancer and is under evaluation in ongoing TNBC clinical trials. Overall, *in vitro* and *in vivo* data suggest that vitamin C sensitizes TNBC to buparlisib treatment.

The improved response of TNBC to buparlisib by vitamin C is unlikely to be caused by its property as an antioxidant since GSH has no effect on the efficacy of buparlisib. Recently, high doses of vitamin C have been shown to inhibit PI3K/AKT pathway in treating thyroid cancer by elevation of reactive oxygen species (ROS) 36. We found that 1,000 μM vitamin C, which can be achieved *in vivo* by intravenous or intraperitoneal injections, indeed increased ROS in BT20 cells. In contrast, 100 μM vitamin C, which can be reached *in vivo* by oral delivery as performed in this study, did not change ROS compared to the baseline (Figure S7), suggesting that ROS is unlikely to play a key role in the sensitization of TNBC to PI3K inhibitors by oral vitamin C. Rather, the impact of vitamin C is likely due to its role in epigenetic regulation, particularly its enhancement of KDM5-mediated histone H3K4 demethylation. Previously, Spangle *et al*. have presented an innovative epigenetic mechanism that links PI3K/AKT signaling to H3K4me3 in breast cancer cells 30. The phosphorylation of KDM5A by AKT is shown to block its translocation to the nucleus, which results in high H3K4me3 and promotes transcription of genes associated with poor clinical outcomes. AKT inhibitors increase KDM5A in the nucleus by blocking its phosphorylation. Our experiments show that PI3K inhibitor buparlisib, like AKT inhibitors, increases the presence of KDM5A in the nucleus. Vitamin C also slightly increases nuclear KDM5A, likely due to the downregulated AKT2 by vitamin C alone. As one member of JmjC domain-containing histone demethylases, KDM5A specifically antagonizes histone H3K4. Vitamin C promotes histone demethylation catalyzed by JmjC domain-containing histone demethylases, which has only been shown previously in stem cells 19, 20. To our knowledge, this is the first time that it has been shown that vitamin C alone decreases histone methylation in non-stem cells. Combination treatment with buparlisib and vitamin C diminished H3K4me3, which is likely a result of the cooperatively enhanced H3K4 demethylation by buparlisib and vitamin C through increasing the presence of KDM5 in the nucleus and serving as a cofactor to promote the enzymatic activity of KDM5.

Vitamin C significantly shifted the transcriptome of *PIK3CA*-mutated BT20 cells. The pathway analysis of RNA-seq data indicates downregulation of the PI3K pathway by vitamin C. Prominently, vitamin C inhibits the expression of PI3K pathway genes, such as AKT2, mTOR, GSK3α, and mLST8. Thus, vitamin C and buparlisib coordinately block PI3K signaling by decreasing PI3K downstream proteins and by inhibiting PI3K enzymatic activity. Furthermore, the altered expression of these PI3K pathway genes by vitamin C appears to be independent of TETs, but dependent on KDM5. Consequently, the improved response of TNBC cells to buparlisib by vitamin C appears to be mediated by KDM5. Additionally, genes that are critical to cancer growth and metastasis, such as AURKB, PCNA, fascin, and FILIP1L, may also play a role in the cooperative anti-tumor activity of vitamin C and buparlisib. Previous studies have shown that H3K4me3 reduction underlies the decreased expression of AURKB, PCNA and other genes 30. Our results suggest that KDM5-mediated H3K4 demethylation likely underpins the upregulation of FILIP1L, and TETs-mediated DNA demethylation could be responsible for the downregulation of fascin by vitamin C and buparlisib. Overall, vitamin C sensitizes TNBC cells to PI3K inhibition mainly by cooperatively promoting KDM5-mediated H3K4 demethylation, which further affect the expression of genes in the P3K pathway and genes relevant to cancer growth and metastasis.

Vitamin C is a safe, well-tolerated micronutrient that is easily accessible and could be conveniently utilized in patient care. Through co-administration with vitamin C at well-tolerated non-toxic doses, lower doses of PI3K inhibitors could be used to achieve an increased therapeutic index, which will also translate to a reduced side effect profile in patients. Beyond TNBC, further studies on the role of vitamin C in PI3K inhibitors in treating HR+ breast cancer in combination with endocrine therapy, and in treating HER2+ breast cancer in combination with anti-HER2 therapy are warranted.

In conclusion, our study suggests that vitamin C expands the therapeutic window of PI3K inhibitor buparlisib in treating *PIK3CA*-mutated TNBC by promoting KDM5-mediated H3K4 demethylation to suppress the expression of PI3K pathway genes and other genes relevant to cancer growth and metastasis. A therapeutic strategy of combining vitamin C with PI3K inhibitors might provide a new opportunity to expand the utility of these promising yet low responsive, toxic cancer drugs for patient care.

## Supplementary Material

Supplementary figures and tables.

## Abbreviations

AURKB: Aurora Kinase B
FILIP1L: Filamin A interacting protein 1-like
GSH: Glutathione
HER2: human epidermal growth factor receptor 2
HR: hormone receptor
i.p.: Intraperitoneal injection
mLST8: Target of rapamycin complex subunit 8
NSG: NOD scid gamma
pAKT: Phosphorylated AKT
PCNA: Proliferating cell nuclear antigen
SVCT2: Sodium-dependent vitamin C transporter 2
TET: Ten-eleven translocation methylcytosine dioxygenases
TNBC: Triple negative breast cancer
TUNEL: Terminal deoxynucleotidyl transferase (TdT)- mediated dUTP nick end labeling
