# Supplementary material for: Vitamin C sensitizes triple negative breast cancer to PI3K inhibition therapy
Source: Theranostics. 2021 Jan 20;11(8):3552–64. doi: 10.7150/thno.53225 (PMC7914363; doi:10.7150/thno.53225)
Supplement: Supplementary file 1 — Supplementary figures and tables. [file thnov11p3552s1.pdf]

Vitamin C sensitizes triple negative breast cancer to PI3K inhibition therapy

Sushmita Mustafi[1], Vladimir Camarena[1], Rehana Qureshi[2], David W. Sant[1], Zachary Wilkes[1], Daniel Bilbao[2], Joyce Slingerland[2,3], Susan B. Kesmodel[2], Gaofeng Wang[1,2]

1. John P. Hussman Institute for Human Genomics, Dr. John T. Macdonald Foundation Department of Human Genetics, University of Miami Miller School of Medicine, Miami, Florida.
2. Sylvester Comprehensive Cancer Center, University of Miami Miller School of Medicine, Miami, Florida.
3. Braman Family Breast Cancer Institute at Sylvester, University of Miami Miller School of Medicine, Miami, Florida.

Corresponding author: Gaofeng Wang, Ph.D., BRB 608, 1501 NW 10th Ave, University of Miami Miller School of Medicine, Miami, FL 33136, USA. E-mail: gwang@med.miami.edu.

Inventory of Supplementary information:

Supplementary Figures **S1-S7**

Supplementary Tables **S1**

Supplementary figures

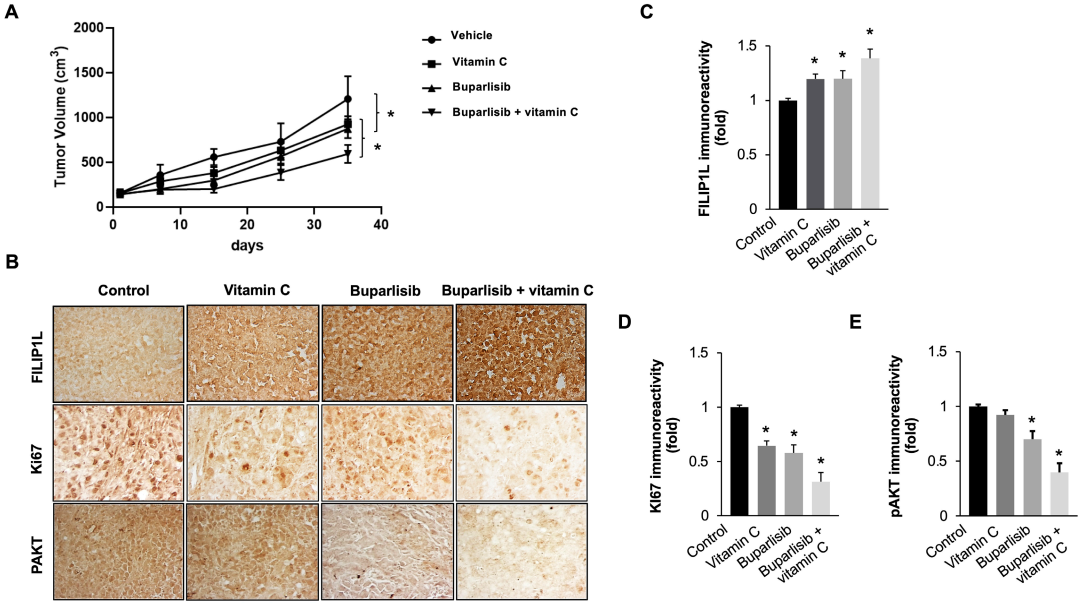

Figure S1: Vitamin C and buparlisib combination treatment of TNBC xenografts. (**A**) Xenograft growth is slower in buparlisib and vitamin C combination group compared to vehicle group or compared to buparlisib alone group (* *P* < 0.05). Tumor volumes were measured by caliper. (**B-E**) Immunostaining and semi-quantification show that buparlisib and vitamin C combination treatment increases FILIP1L, but decreases Ki-67 and pAKT in TNBC xenografts.

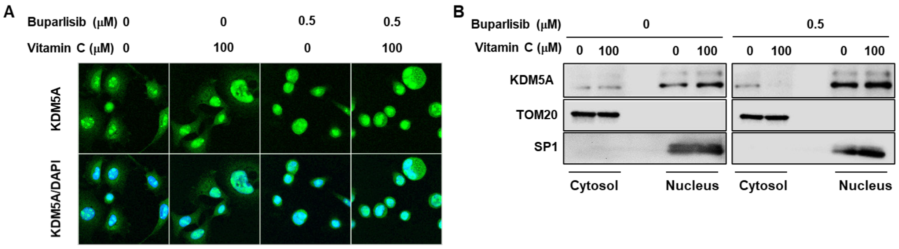

Figure S2: Buparlisib promotes KDM5A nuclear translocation. (**A**) Buparlisib obviously, while vitamin C slightly, increases the presence of KDM5A in the nucleus of BT20 cells, has no significant effects, shown by immunofluorescence. (**B**) Western blot of cellular fractions shows that co-treatment with buparlisib and vitamin C markedly promote KDM5A nuclear translocation. TOM20 was used as a cytosol marker and SP1 a nuclear marker.

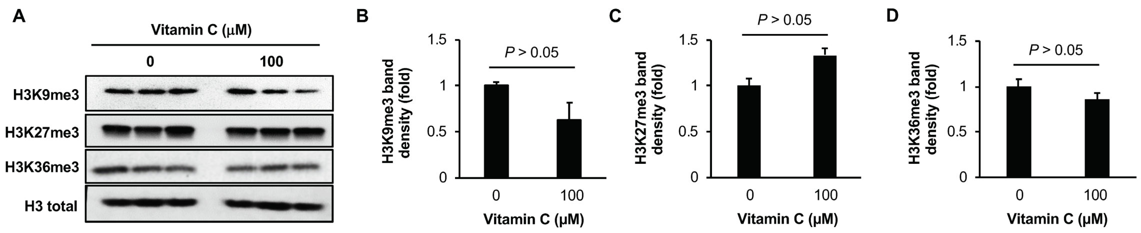

Figure S3: Vitamin C does not change trimethylation at H3K9, H3K27 and H3K36. (**A**) Western blot of H3K9me3, H3K27me3 and H3K36me3 in BT20 cells treated with or without vitamin C (100 μM). (**B**) Semi-quantification of band density shows that vitamin C does not significantly change H3K9me3. (**C**) Semi-quantification of band density shows that vitamin C does not significantly change H3K27me3. (**D**) Semi-quantification of band density shows that vitamin C does not significantly change H3K36me3.

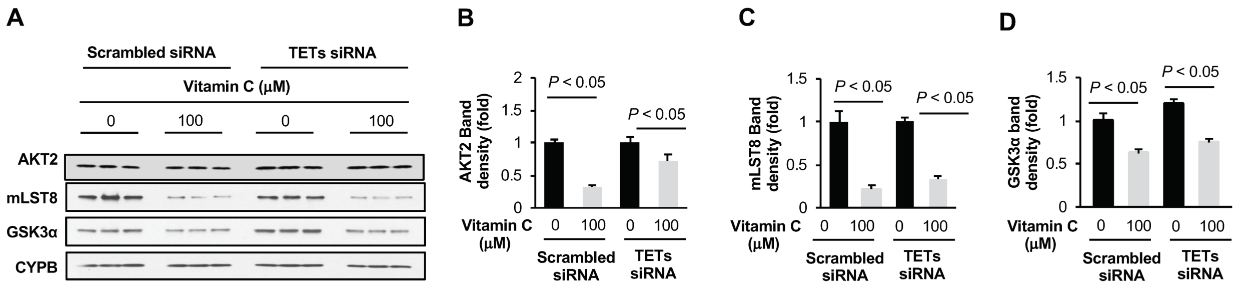

Figure S4. Silence of TETs has no impact on the suppression of PI3K pathway genes by vitamin C. A) Western blot of AKT2, mLST8 and GSK3α in BT20 cells treated with either scrambled siRNA or siRNA targeting TETs, followed by vitamin C (100 μM). (**B**) Semi-quantification of band density shows that Vitamin C continues to decrease AKT2 in BT20 cells after TETs knockdown. (**C**) Semi-quantification of band density shows that Vitamin C continues to decrease mLST8 in BT20 cells after TETs knockdown. (**D**) Semi-quantification of band density shows that Vitamin C continues to decrease GSK3α in BT20 cells after TETs knockdown.

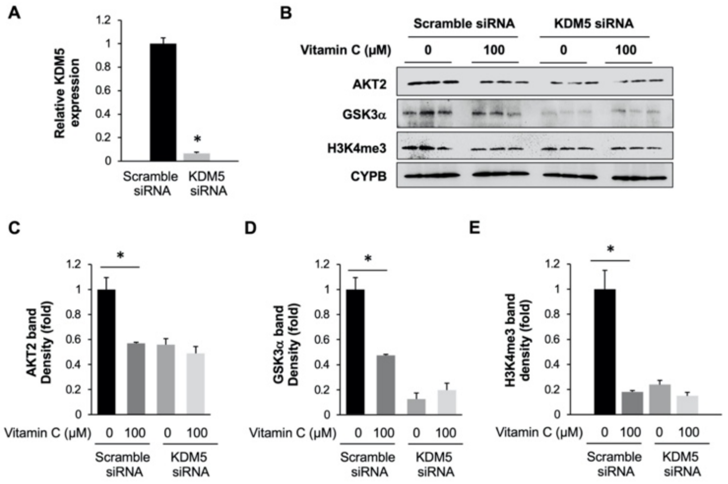

Figure S5: Knocking down KDM5 abolishes the effect of vitamin C on PI3K pathway genes. (**A**) The knock down of KDM5 in BT20 cells by siRNA is verified by qRT-PCR . (**B-E**) Western blot and semi-quantification show that vitamin C decreases AKT2, GSKα and H3K4me3 in scramble siRNA group but not in KDM5 siRNA group.

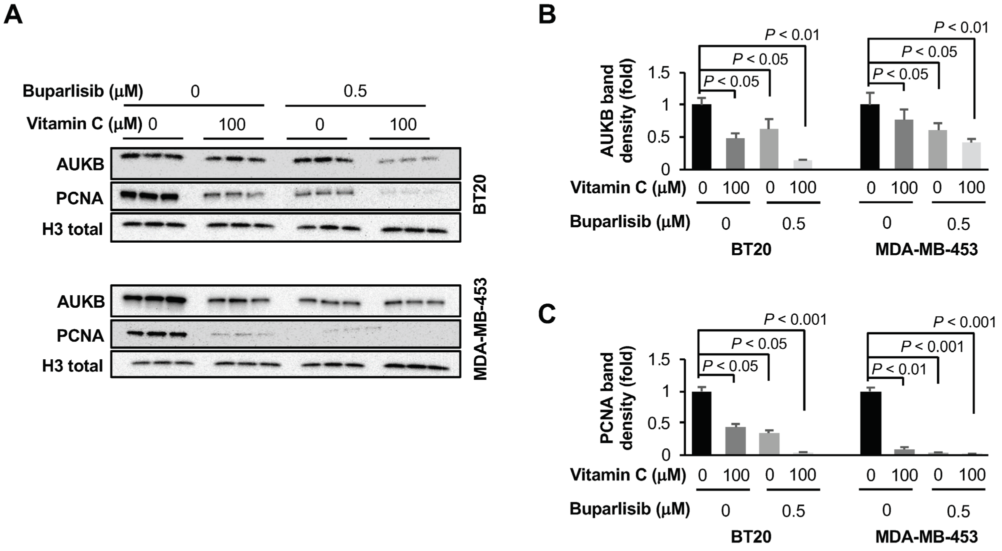

Figure S6. Vitamin C and buparlisib cooperatively decrease the expression of genes critical to cancer growth. (**A**) Western blot of AUKB and PCNA in BT20 cells treated with vitamin C and buparlisib. (**B**) Semi-quantification of band density shows that vitamin C (100 μM) alone or buparlisib (0.5 μM) alone reduces AUKB in BT20. Co-treatment with vitamin C and buparlisib further decreases AUKB. (**C**) Semi-quantification of band density shows that vitamin C (100 μM) alone or buparlisib (0.5 μM) alone reduces PCNA in BT20. Co-treatment with vitamin C and buparlisib further decreases PCNA.

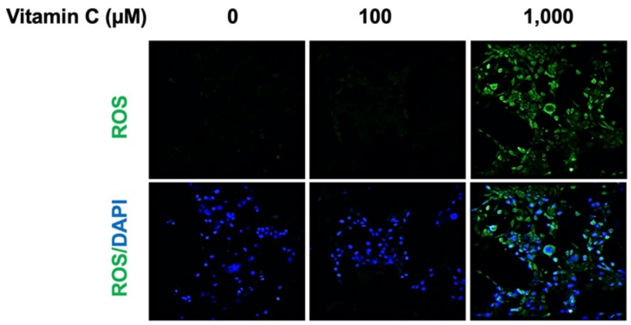

Figure S7: ROS induction by high-dose vitamin C. Compared to the baseline, only high-dose vitamin C (1,000 μM), but not the low-dose vitamin C (100 μM), increases ROS in BT20 cells.

Supplementary table

Table S1. The altered transcription of genes relevant to metastasis by vitamin C

Gene symbol	Gene name	Fold change	P value
FILIP1L	Filamin A Interacting Protein 1 Like	3.167279208	0.017421485
PDE4D	Phosphodiesterase 4D	2.485549801	1.76E-07
FARS2	Phenylalanyl-TRNA Synthetase 2, Mitochondrial	2.290263148	2.89E-06
NR4A3	Nuclear Receptor Subfamily 4 Group A Member 3	2.057003316	0.000612926
PPP1R12B	Protein Phosphatase 1 Regulatory Subunit 12B	1.914992189	5.01E-07
RBPMS	RNA Binding Protein, MRNA Processing Factor	1.784208227	2.65E-07
ZFAND3	Zinc Finger AN1-Type Containing 3	1.65843867	0.000332419
DIO2	Iodothyronine Deiodinase 2	1.594849544	3.47E-05
LYPLA1	Lysophospholipase 1	1.442980363	0.000225297
NEIL3	Nei Like DNA Glycosylase 3	1.437086168	0.001539343
BMPR1A	Bone Morphogenetic Protein Receptor Type 1A	1.38192327	0.025393425
JUND	Jun-D	0.738785426	0.001450741
GSN	Gelsolin	0.732593025	0.001447461
LIG1	DNA Ligase 1	0.729078767	0.004417261
DDX17	DEAD-Box Helicase 17	0.724176009	0.001609957
KRT15	Keratin 15	0.721074539	0.002739526
POLD1	DNA Polymerase Delta 1	0.718609553	0.011252718
FBLN1	Fibulin 1	0.714137576	0.001314202
SEMA4A	Semaphorin 4A	0.711407132	0.016627965
TNPO2	Transportin 2	0.695780858	0.00414253
DNAJB1	DnaJ Heat Shock Protein Family (Hsp40) Member B1	0.688428568	0.000235078
GADD45B	Growth Arrest And DNA Damage Inducible Beta	0.682338723	0.001108662
ATXN2L	Ataxin 2 Like	0.65910411	3.45E-05
ZFP36L1	ZFP36 Ring Finger Protein Like 1	0.644570422	0.000862215
HIPK1	Homeodomain Interacting Protein Kinase 1	0.628141835	0.001250858
LRRC47	Leucine Rich Repeat Containing 47	0.625350102	5.30E-05
ABCA2	ATP Binding Cassette Subfamily A Member 2	0.623986104	0.000302561
C15orf39	Chromosome 15 Open Reading Frame 39	0.617907756	3.41E-06
TAX1BP3	Tax1 Binding Protein 3	0.608439549	0.021030155
EIF3A	Eukaryotic translation initiation factor 3 subunit A	0.590436903	3.08E-06
RAB26	RAB26, Member RAS Oncogene Family	0.590380107	0.00390852
HECTD3	HECT Domain E3 Ubiquitin Protein Ligase 3	0.575841789	2.56E-05
MVP	Major Vault Protein	0.542541782	1.56E-09
FUCA1	Alpha-L-Fucosidase 1	0.523724789	0.002277139
TRIM28	Tripartite Motif Containing 28	0.510131427	2.10E-08
FSCN1	Fascin Actin-Bundling Protein 1	0.505392484	0.01437903
